# Physics-informed neural network reconciles Australian displacements and tectonic stresses

**DOI:** 10.1038/s41598-023-50759-0

**Published:** 2023-12-28

**Authors:** Thomas Poulet, Pouria Behnoudfar

**Affiliations:** Commonwealth Scientific and Industrial Research Organisation (CSIRO) Mineral Resources, Kensington, Perth, WA 6151 Australia

**Keywords:** Geodynamics, Tectonics

## Abstract

Stress orientation information is invaluable to evaluate active tectonic forces within the Earth’s crust. The global dataset provided by the World Stress Map offers a rich resource of stress indicators, facilitating the calibration of mechanical models to extract complete stress and displacement fields. However, traditional inversion processes are hampered by the manual tuning of geomechanical properties and boundary conditions to reconcile simulations with observations. In this study, we introduce ML-SEISMIC (machine learning for stress estimation integrating satellite image and computational modelling), a physics-informed deep neural network approach to autonomously align stress orientation data with an elastic model. It nearly completely bypasses the need for explicit boundary condition inputs and yields comprehensive distributions of material properties, displacements, and stress tensors. Application of this methodology to Australia, coupled with precise global navigation satellite systems observations, unveils a robust and scale-independent interpolation framework. Additionally, it pinpoints regions where stress orientation reinterpretation is warranted. Our results present a streamlined yet powerful process, offering a substantial leap forward in geodynamic investigations. This approach promises to unify velocity and stress orientation observations with physical models, ushering in a new era of insights into Earth’s dynamic processes.

## Introduction

**Figure 1:**
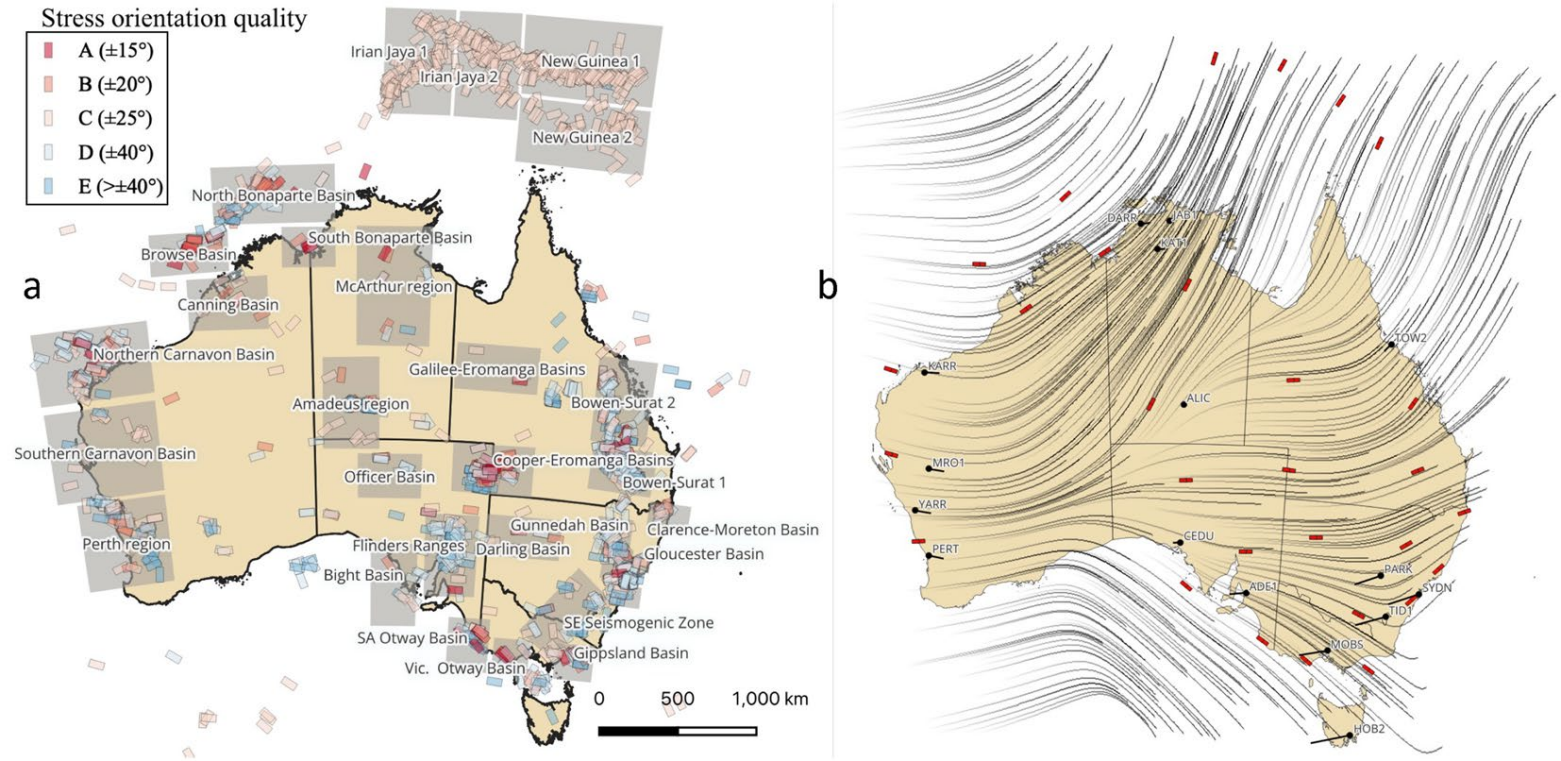
(**a**) Maximum horizontal compressional stress $S_{Hmax}$ orientation from the World Stress Map in Australia and Papua New Guinea^5^, coloured by quality, and locations of 30 main stress provinces (grey rectangles); (**b**) average stress orientations per province (red markers) and corresponding stress orientation obtained by kriging (grey traces).

The current stress state of the Earth’s crust plays a critical role in numerous geological applications including carbon or hydrogen storage, nuclear waste disposal, reservoir engineering, tunnel or mine stability, fault reactivation, or borehole planning, to name a few. The full stress information must therefore be assessed as accurately as possible, yet it is rarely measured directly but instead often inverted exploiting observations from faults^1^, seismic data^2^, or boreholes and rock cores^3^ for instance. The largest source of current-day crustal stress information is arguably the World Stress Map (WSM)^4^, which gathers tens of thousands of records globally and provides in particular some estimates of the maximum horizontal stress orientation. Overall, Australia is one of the best-studied areas globally and its latest present-day stress field estimate^5^ builds on the regular improvements of the WSM information to refine the picture from previous studies^6–9^. The WSM database associates quality information with each stress orientation data. Still, even the most accurate (A-quality) records carry an estimated error of $\pm 15^{\circ }$, making some interpretation necessary when selecting which data to consider to build a map. For the Australian case, the high concentration of information in localised areas makes it possible to derive relatively reliable average stress orientation (Fig. 1a) in the main stress provinces^5^, which then allows to derive a stress orientation map through geostatistics for instance (Fig. 1b).

Such an approach, however, only provides a subset of the complete stress information, namely the horizontal stress orientation. In particular, it does not ensure the physical consistency of the generated map, as the methodology remains purely geostatistical in nature. Independent geomechanical analyses are then required to generate models that respect physical laws, but which then reconcile the stress orientation observations with various levels of accuracy (see references and assessments in^10^). The best-fit geomechanical model to date for Australia^10^ was obtained by manually fine-tuning the material properties of a three-dimensional elastic model to specifically match the stress orientation information as well as possible. Despite the good quality results, that trial-and-error methodology cannot be suggested as a template for any selected area globally, principally because of the particularly intensive fitting process required and the dependency of the results on the choice of boundary conditions, inferred in that study from the Indo-Australian plate tectonic settings as constant displacements on the edges of a pentagon model encompassing Australia.

Recently, physics-informed neural networks (PINNs) have been specifically developed to tackle the problem of solving multi-physics problems governed by partial differential equations (PDEs), both for forward and inverse problems^11^. These problems include solving linear elastic solid-mechanics^12^—the type commonly used for stress inversion, for example, in Australia^5,13^. Such models, however, are used mainly in conditions close to those of traditional forward simulators, i.e. for well-posed problems with appropriate boundary conditions. The purpose of this study is to introduce a machine-learning approach to retrieve the full stress and displacement fields from the governing physical equations, satellite data, and limited stress-orientation measurements. Due to the lack of boundary conditions associated with the PDEs, the problem is ill-posed and to overcome this issue, our novel approach takes into account the eigenvalues of the approximated stress field and optimizes them with respect to the stress orientations. We apply the proposed technique to estimate the stress field and displacement in the Australian continent and retrieve the corresponding effective material properties.

We use our machine learning approach to approximate a 2D linear elastic problem, by optimising displacement, stress field, and elastic properties through the definition of a loss function that enforces the momentum balance, constitutive relationships between the Cauchy stress tensor and the infinitesimal strain tensor, and the small strain definition (“Methods” section). Furthermore, we include the match of stress orientation information in the loss function.

## Geomechanical models with minimal boundary conditions

**Figure 2:**
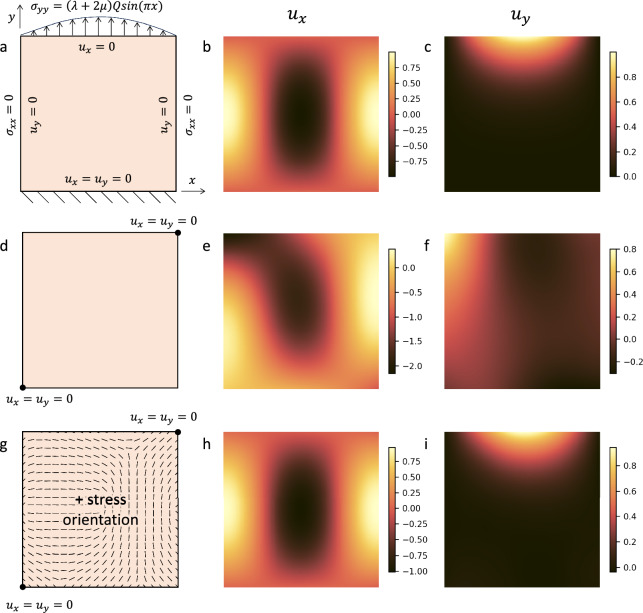
Benchmark results. The top row displays (**a**) the model setup over $[0,1]^2$ with all edges subject to stress or displacement boundary conditions^12^ and the corresponding analytical solution for the resulting displacement fields $u_x$ (b) and $u_y$ (c). The second row indicates the results obtained by commonly used PINNS^12^ on a similar scenario with only two displacement constraints in opposite corners. The resulting displacements (e,f) differ greatly from the analytical solution. The bottom row shows (g) a varying setup with only two displacement constraints in opposite corners, but with stress orientation constraints over the domain, leading to qualitatively identical results for $u_x$ (h) and $u_y$ (i). These results highlight the validity of our method and the fact that stress orientation can alleviate the lack of boundary conditions.

First, we evaluate our approach to a problem with an analytical solution. The original problem consists of a 2D square with displacement and stress boundary conditions shown in Fig. 2a, with a specific body force applied everywhere (see Supplementary Information). We solve instead a modified version of the problem, where we remove all stress boundary conditions and keep zero displacement boundary conditions at two points only, here the bottom-right and top-left corners, to enforce a unique solution since the problem is defined within a constant stress or rigid deformation. This modification of the boundary conditions (Fig. 2d) naturally leads to a different solution (Fig. 2e,f).

We then add the stress orientation information as constraints on 400 regularly spaced collocation points (Fig. 2g). The results show that we retrieve the displacement fields $u_x$ and $u_y$ (Fig. 2h,i) matching the analytical solution (Fig. 2b,c, see Supplementary Information for quantitative comparisons), i.e. that constraints on the stress orientation can alleviate the lack of boundary conditions, which can be retrieved automatically.

## Retrieving Australian stress tensors, using stress orientation or satellite observations?

Building on the findings from the previous section, we now consider an application to the Australian continent to recover the displacement and stress distributions, exploiting an effective elastic model, constrained by the stress orientation and velocity information available (Fig. 1b). We consider the NASA Global Navigation Satellite System (GNSS) time series^14^ with 16 stations available in Australia (Fig. 1b). The measured velocity vectors are translated by a constant to set the value to zero in Alice Springs (ALIC station), at the centre of the model. They are then rescaled for normalisation purposes, without any side-effect to the selected mechanical model. Note that those time series record accurate and nearly constant horizontal velocities for all 16 stations over the last 25 years, with an error below 1% for all observations (0.27% on average). Therefore, we design SEISMIC-ML for steady-state conditions.

There is a notable difference between the previous benchmark and its analytical solution, since only the displacement observations are very accurate, while the stress orientations are based on estimates with much larger uncertainty ($\pm 25^{\circ }$ at best, see Fig. 1a) and some averaging not accounting for local stress variations. Given the explicit dependency between stress and displacement from the constitutive model (“Methods”, Fig. 1), all corresponding observations must be geomechanically consistent for the numerical optimisation to converge, yet this is not assured by default with the observations. This leads us to consider the two end-members scenarios, one where the inversion is performed mainly on the stress orientations and the other where only the velocity data is used for observations.

**Figure 3:**
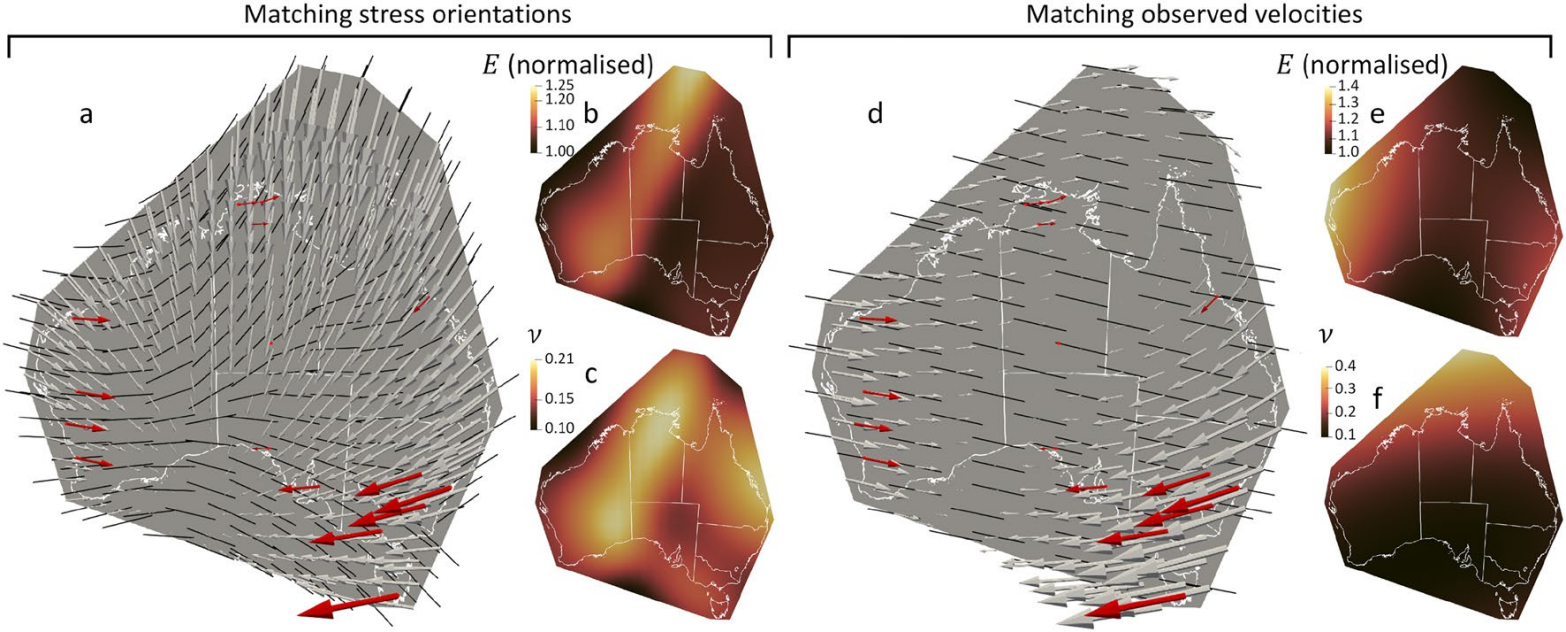
End-member scenarios when (i) matching the interpolated stress orientations of Fig. 1b at all collocation points (**a**–**c**) or (ii) matching the 16 velocity observations (**d**–**f**). The resulting displacements (grey arrows) and stress orientations (black dashes) are shown in subfigures (**a**) and (**c**), along with observed velocities (red arrows) for comparison. The corresponding distributions of material properties—normalised Young’s modulus *E* and Poisson ratio $\nu$—are shown in subfigures (**b**,**c**) for the first scenario and subfigures (**e**,**f**) for the second one. Matching all stress orientations leads to some mismatch for the displacements while matching the displacements leads to a nearly constant stress orientation.

Firstly, we consider the Australian stress map of Fig. 1b, without any boundary conditions information. We use the GDA 2020 Australian Albers (EPSG 9473) projection, an equal area conic projection centred on mainland Australia, in order to minimise the projection effects on the solid mechanics problem. We take as constraints the 30 average stress province orientations from published data^5^, within an arbitrarily drawn convex polygon including our Australian area of interest (Fig. 3a). We use a geostatistical interpolation method—kriging^15^, as one of the most used techniques—to evaluate the stress orientation observations at every collocation point (Fig. 1b). As indicated in the previous benchmark, two displacements are needed and we select the rescaled observed velocities in Perth and Sydney (PERT and SYDN stations on FigurFig. 1b) as displacement constraints in the loss function. All other components of the loss function are set on every collocation point to enforce the momentum balance, constitutive model, and small strain definition. This conserves all properties within the numerical precision of the algorithm. The two elastic parameters needed can be defined in various manners, through the Lamé coefficients, or typically through the Young’s modulus ($E=\mu \frac{3\lambda +2\mu }{\lambda +\mu }$) and Poisson ratio ($\nu =\frac{\lambda }{2(\lambda +\mu )}$). We define *E* and $\nu$ as functionals over the input (*x*, *y*) and normalise *E*. The results show that we retrieve a stress field matching the stress orientation constraints, for some distributions of Young’s modulus and Poisson ratio (Fig. 3a–c). The corresponding displacement field, however, displays notable differences with the velocity observations.

Secondly, we run the optimisation based on the 16 velocity observations, without any stress orientation constraints. The results show (Fig. 3d–f) that all velocities can be matched by a geomechanical model, in which the stress orientation is nearly constant in the west-northwest—east southeast direction (Fig. 3d). This indicates a single effective stress orientation for Australia capturing the overall displacement distribution.

## Matching satellite observations while identifying stress orientation discrepancies

**Figure 4:**
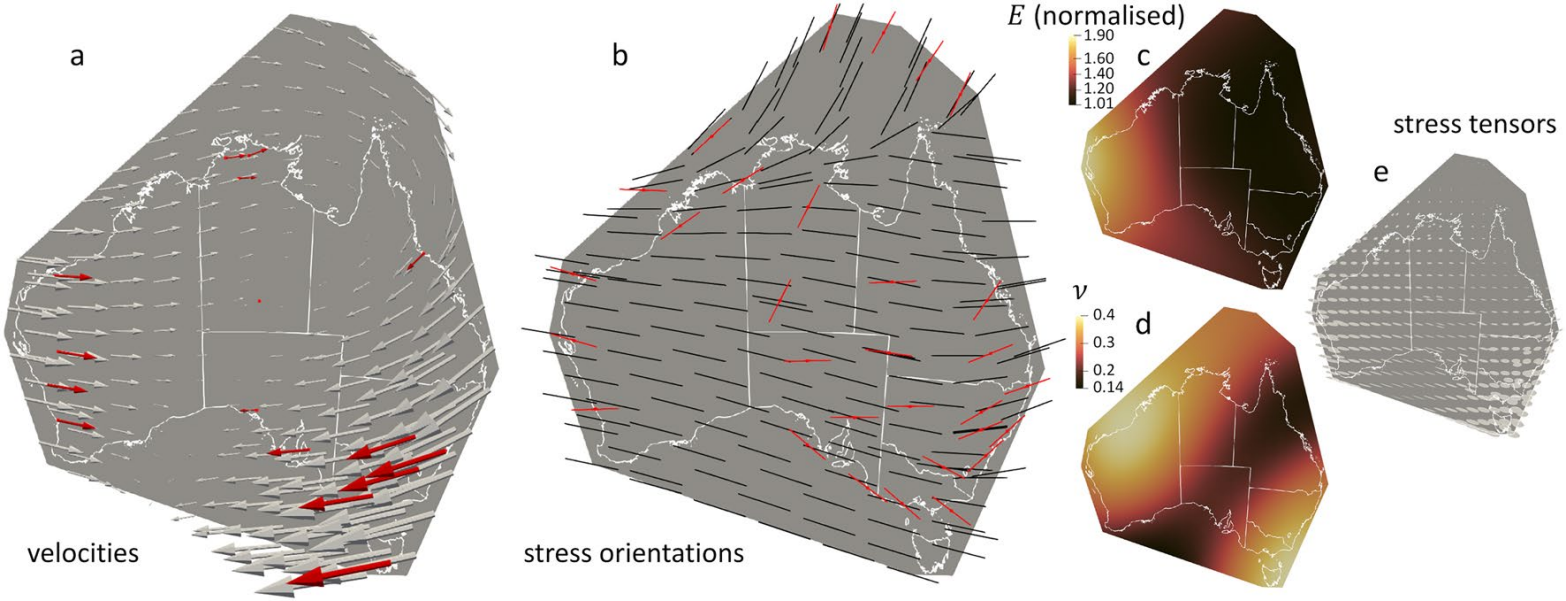
Results for scenario accounting for the 30 non-interpolated stress orientations and 16 observed velocities of Fig. 1. (**a**) Displacements (grey arrows) match very well all observed velocities (red arrows). (**b**) The stress orientations (black dashes) match most of the input, with some notable discrepancies. The other subfigures show the corresponding distributions of (**c**) normalised Young’s modulus *E*, (**d**) Poisson ratio $\nu$, and (**e**) stress tensors (grey ellipses).

The final step consists of considering simultaneously all velocity and stress observations. To account for potential inconsistencies, the velocity observations are prioritised over the stress orientations by setting a factor $\gamma =10^{-2}$ (Eq. 3) to the stress orientation component of the loss function. The results show that we recover, as expected, a displacement field matching perfectly the velocity observations (Fig. 4a) and reasonably well the stress orientation data (Fig. 4b).

Our approach contrasts with traditional geomechanical studies^10,13^ where the material properties are set manually from the extensive literature review as best guesses and where boundary conditions also need to be postulated by trial-and-error. Instead, our fit is obtained automatically, without stipulating any boundary conditions or material properties. The resulting distribution of Young’s modulus (Fig. 4c) shows an increased rock strength from the East Coast to the West Coast, in agreement with the variation of lithosphere thickness with age across Australia, thinner for the Phanerozoic (< 542 Ma) on the East Coast than for the Proterozoic (542–2500 Ma) and Archean (> 2500 Ma) in the central and western parts of the continent^16^. This also matches the fact that the western and central parts of Australia contain all the cratons, which are mechanically stronger than the fold belts located on the East Coast^13^. The optimisation additionally leads to the distribution of Poisson ratio (Fig. 4d) and allows to get the full stress information (Fig. 4e) from observations only, without any bias as there are no user assumptions involved.

Having removed any possibility of introducing artificial errors from the choice of boundary conditions and distribution of material properties, the discrepancies in stress orientation (Fig. 4b) indicate that the stress input data in those areas are simply not optimal at the selected scale of interest. This could be due to various factors, including the original data uncertainty, the averaging of all stress orientations, or the lack of observations in important areas. Fortunately, our methodology can easily include additional data at multiple scales, depending on the efficiency of the machine learning solution selected, which places the challenge mostly on the reliability of the stress orientation observations.

## ML-SEISMIC’s contribution: displacement patterns and stress tensor analysis

Our approach determines as output the displacements at the edges of the model, which are naturally consistent with the constraints imposed regardless of the geometry selected, and could then be used in turn as boundary conditions for further geomechanical models (e.g. for 3D models). Here, we automatically retrieve the displacement patterns around Australia responsible for the overall compressive state of the continent (Fig. 4a) and the results (Fig. 4) remind us that displacements are not necessarily aligned with the maximum compressive stress direction, as seen for instance on the southwestern part of the model, where displacements don’t intuitively match the expected ridge-push force from the mid-ocean ridge between the Antarctic and Indo-Australasian plates^10,13^. We also retrieve the distribution of stress tensors (Fig. 4e), which can be used in slip-tendency analyses for fault reactivation^17^, or in fault dilation tendency studies^18^ in cases where fluid flow is a key concern, as in geothermal energy^19^, carbon sequestration^20^ or hydrogen storage^21^, for instance. ML-SEISMIC takes into account GNSS observations not only to show that large-scale averaged stress orientations must be revisited but also to identify where (Fig.4a).

The findings presented in this study contribute to the advancement of our comprehension of tectonics and offer a valuable complement to uncertainty quantification analyses for geomechanical models^22^ or geostatistical investigations that reconcile stress orientation and displacements^23^. It is noteworthy that our methodology is highly adaptable and applicable across a wide range of scales, spanning from crystallographic investigations^24^ to continental-scale analyses^25,26^. Importantly, the versatility of our approach holds significant promise, particularly due to its ease of extension. Previous applications of Physics-Informed Neural Networks (PINNs) have already demonstrated their proficiency in addressing three-dimensional elastic problems^27^, plasticity modelling^28,29^, handling discontinuities^30^, and resolving multi-scale challenges^31^. We anticipate this study to serve as a catalyst for a multitude of forthcoming scientific inquiries, further advancing our understanding of complex geological and tectonic phenomena.

## Methods

We introduce a new approach to estimate the stress field on a physical domain $\Omega$ using a deep learning class of physics informed neural networks (PINNs). PINNs represent indeed a suitable machine-learning strategy for this type of physics. Considering the momentum balance, the constitutive relationship between the Cauchy stress tensor ($\sigma$) and the infinitesimal strain tensor ($\varepsilon$), as well as the small strain definition linking the strain tensor with the displacement vector *u*, the equations of linear elasticity read:

1 $$\begin{aligned} \begin{aligned} \sigma _{ij,j} + f_i&=0, \\ \sigma _{ij}&= \lambda \delta _{ij}\varepsilon _{kk}+2\mu \varepsilon _{ij}, \\ \varepsilon _{ij}&= \frac{1}{2} \left( u_{i,j} + u_{j,i} \right) , \end{aligned} \end{aligned}$$

where the vector *f* denotes a body force, $\lambda$ and $\mu$ the Lamé coefficients, and $\delta$ the Kronecker delta. Here, Einstein’s notation is used. Equation (1) is well-posed with provided boundary conditions. We propose an approach to deploy neural networks (NNs) for solving Eq. (1) in a 2D domain in the absence of boundary conditions. Furthermore, we introduce two constraints to the system considering the measured displacement vectors ($u^*_x,\, u^*_y$) provided by GNSS data, and $\theta ^*$ being the measured stress orientation. That is, we approximate Eq. (1) by a deep neural network which takes the spatial coordinates and available displacement observations as inputs to predict the corresponding displacement vector and stress tensor, i.e., $(x , y, u^*_x,u^*_y,\theta ^*) \rightarrow ( u_x , u_y, \sigma _{xx}, \sigma _{xy}, \sigma _{yy})$. For the activation function, we use the nonlinear function $\phi (\cdot )=\tanh (\cdot )$. Our NN includes *l* layers with $n_i$ neurons in each layer and delivers the solution

2 $$\begin{aligned} u_{NN}(\varvec{x};\,\varvec{W},\,\varvec{b})=G \circ \mathcal {N}^{(l)}\circ \mathcal {N}^{(l-1)} \circ \cdots \circ \mathcal {N}^{(1)}(\varvec{x}), \end{aligned}$$

with *G* a linear mapping acting on the last layer. In each hidden layer *i*, the nonlinear mapping is $\mathcal {N}^{(i)}(\cdot )=\phi (\varvec{W}_i\times \cdot +\varvec{b}_i)$. We deploy four hidden layers with 40 neurons each and a batch size of 32 for the ADAM^32^ optimiser. The learning rate is set as a decreasing function of epochs as required. The choice of network hyper-parameters, i.e., depth, width, activation functions, etc., is based on our numerical experiments and may not be optimal. Proposing the optimal choices is beyond the scope of this paper.

The loss function reads

3 $$\begin{aligned} \begin{aligned} \mathscr{L}&= |u_x-u_x^*|+|u_y-u_y^*| + \gamma |d(\theta ,\theta ^*)| \\&+ |\sigma _{xx,x}+\sigma _{xy,y}+f_x^*| + |\sigma _{xy,x}+\sigma _{yy,y}+f_y^*| \\&+ |(\lambda +2\mu )\varepsilon _{xx}+\lambda \varepsilon _{yy}-\sigma _{xx}| + |(\lambda +2\mu )\varepsilon _{yy}+\lambda \varepsilon _{xx}-\sigma _{yy}| + |2\mu \varepsilon _{xy}-\sigma _{xy}|, \end{aligned} \end{aligned}$$

where $\theta$ is the azimuth of the eigenvector associated with the most negative eigenvalue (maximum compressive stress), |*x*| the mean squared error of the quantity *x* and function $d(\alpha , \beta )$ denotes the smallest angle difference between two lines of azimuths $\alpha$ and $\beta$. We consider $\lambda$ and $\mu$ as network parameters that change during the training phase to identify them at spatial coordinates. We use the weight $\gamma$ as a free parameter to control the relative importance of the stress orientation overall.

All calculations were run on a M1 Macbook Pro with 32 GB of memory. As a conservative indication, obtaining the results shown in Fig. 4 took 9.5 min on a single CPU.

### Supplementary Information

Supplementary Information.

## Data Availability

A Google Colaboratory script used to produce the benchmark results is freely available^33^. The data supporting the findings of this study are available from the corresponding author upon reasonable request.
