# Supplementary material for: Physics-informed neural network reconciles Australian displacements and tectonic stresses
Source: Sci Rep. 2023 Dec 28;13:23095. doi: 10.1038/s41598-023-50759-0 (PMC10754839; doi:10.1038/s41598-023-50759-0)
Supplement: Supplementary file 1 — Supplementary Information. [file 41598_2023_50759_MOESM1_ESM.pdf]

Supplementary Information

Benchmark

The importance of the boundary conditions and the stress orientation is investigated on a problem defined by Equation 1 in the main document, on a square domain of size 1, with the boundary conditions pictured on Figure 1 and the following forcing terms:

$$\begin{aligned} f_x &= \lambda[4\pi^2 cos(2\pi x)sin(\pi y) - \pi cos(\pi x)Qy^3] \\ &\quad + \mu[9\pi^2 cos(2\pi x)sin(\pi y) - \pi cos(\pi x)Qy^3], \\ f_y &= \lambda[-3sin(\pi x)Qy^2 + 2\pi^2 sin(2\pi x)cos(\pi y)] \\ &\quad + \mu[-6sin(\pi x)Qy^2 + 2\pi^2 sin(2\pi x)cos(\pi y) + \pi^2 sin(\pi x)Qy^4/4], \end{aligned} \tag{1}$$

such that the analytical solution reads:

$$\begin{aligned} u_x(x,y) &= cos(2\pi x)sin(\pi y), \\ u_y(x,y) &= sin(\pi x)Qy^4/4. \end{aligned} \tag{2}$$

Diverse permutations of the problem are evaluated with the same physics informed deep neural network through the following models (see first column of Figure 1):

- **case 1** is the reference case considering the full set of boundary conditions (stress and displacement) as constraints, without any stress orientation information. It corresponds to the problem that would be solved by a typical mechanical simulator to obtain the analytical solution.
- **case 2** uses the full set of boundary conditions and stress orientation at every collocation point as constraints.
- **case 3** is a degraded version of case 2, where all stress boundary conditions are removed.
- **case 4** is a version of case 3, where the displacement boundary conditions are absent on all edges of the model except the bottom one.
- **case 5** is like case 4, but displacement boundary conditions are kept only on the two bottom corners of the model.
- **case 6** is a modified version of case 5, where displacement boundary conditions are kept only on the bottom-left and top-right corners of the model.
- **case 7** is similar to case 5 in terms of boundary conditions but without the stress orientation constraints at the collocation points.

The results of the corresponding displacement fields u_x and u_y show (Fig. 1) that removing progressively the boundary conditions still allows retrieving adequate solutions, despite losing some accuracy as the set of constraints on the boundaries is reduced (Table 1). For the purpose of the comparison, no emphasis was placed on optimising the convergence of the neural network and more accurate results could be obtained. While the errors compared to the analytical solution are non-negligible, the main result is that

Table 1: Results comparison between all cases. The relative loss indicates the quality of the convergence for the optimisation process. The absolute and relative errors for u_x and u_y, using L_∞ and L_2 norms, show reasonably good results compared to the analytical solution, except for case 7 which converges to a different solution.

Case	Relative loss (convergence)	Absolute error $\lvert u_x - u_x^{ana} \rvert_\infty$	Absolute error $\lvert u_y - u_y^{ana} \rvert_\infty$	Relative error $\frac{\lvert u_x - u_x^{ana} \rvert_2}{\lvert u_x^{ana} \rvert_2}$	Relative error $\frac{\lvert u_y - u_y^{ana} \rvert_2}{\lvert u_y^{ana} \rvert_2}$
1	2.61×10^{-6}	3.71%	3.58%	1.09%	2.04%
2	2.14×10^{-6}	4.00%	2.57%	1.14%	3.01%
3	3.43×10^{-6}	6.58%	11.57%	3.40%	10.34%
4	2.41×10^{-6}	13.07%	10.31%	7.01%	7.27%
5	3.97×10^{-6}	12.94%	14.47%	7.81%	11.59%
6	2.62×10^{-6}	6.88%	13.54%	3.41%	19.56%
7	2.09×10^{-6}	215.6%	117.6%	165.3%	126.9%

we can retrieve qualitatively the correct solution, when considering the stress orientation, from boundary constraints at only two points. Note that we need displacement constraints at two points for disambiguation purposes, as the problem is defined to be within a constant stress and rigid transformation. Case 7 shows that without stress orientation constraints, the lack of boundary conditions results in another solution satisfying the same system of equations with different boundary conditions.

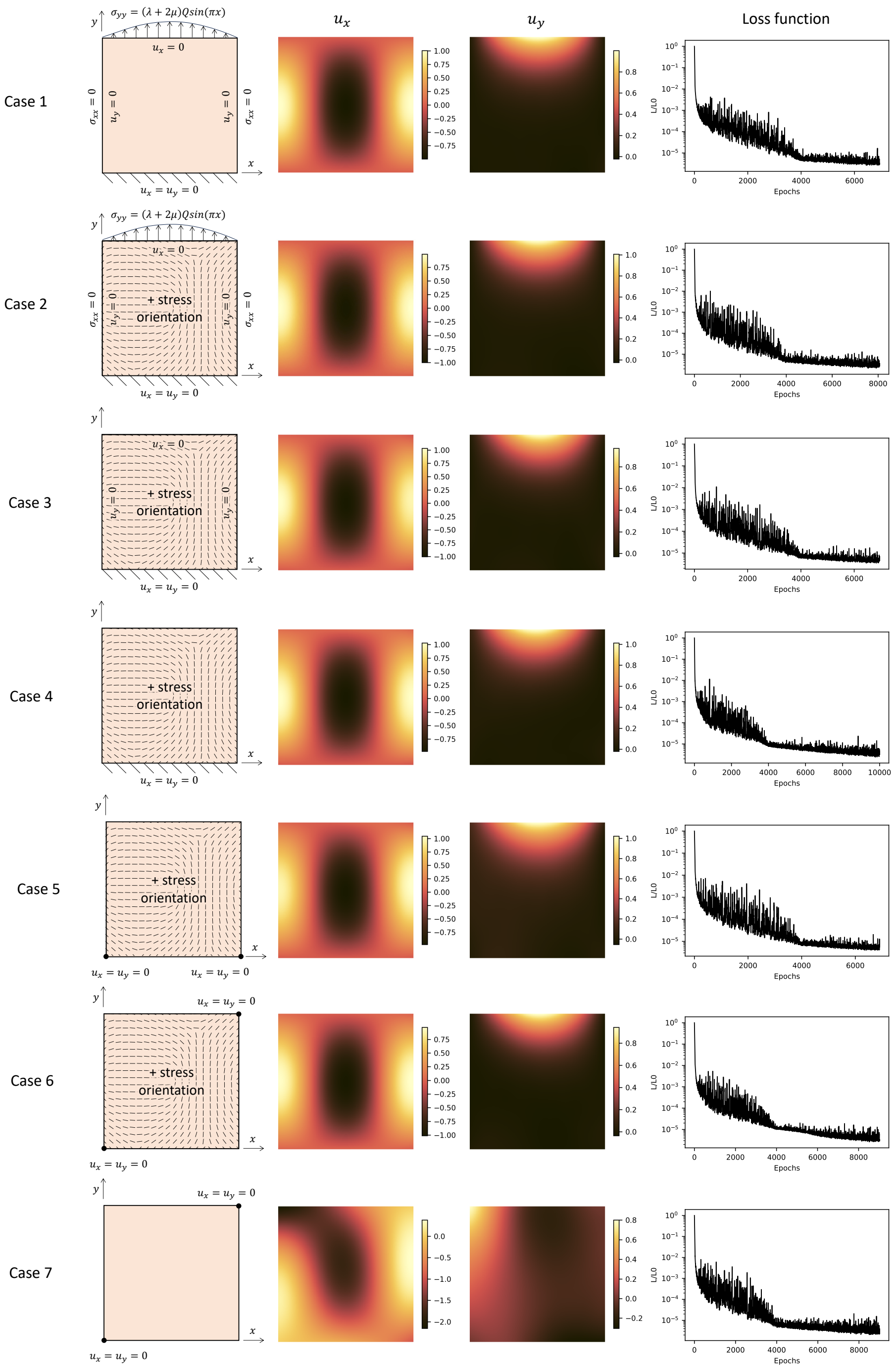

Figure 1: Variation of cases with different boundary constraints, showing the model setup (first column), u_x and u_y fields (second and third columns), as well as the relative loss function (fourth column).
